# Live Cell Imaging Supports a Key Role for Histone Deacetylase as a Molecular Target during Glioblastoma Malignancy Downgrade through Tumor Competence Modulation

**DOI:** 10.1155/2019/9043675

**Published:** 2019-08-08

**Authors:** Aline Menezes, Gustavo Henrique dos Reis, Maria Cecília Oliveira-Nunes, Fernanda Mariath, Mariana Cabanel, Bruno Pontes, Newton Gonçalves Castro, José Marques de Brito, Katia Carneiro

**Affiliations:** ^1^Laboratório de Proliferação e Diferenciação Celular, Instituto de Ciências Biomédicas, Universidade Federal do Rio de Janeiro, Av. Carlos Chagas Filho 373, Bloco F Sala F2-01, Rio de Janeiro 21941-902, Brazil; ^2^Laboratório de Pinças Ópticas, Instituto de Ciências Biomédicas, Universidade Federal do Rio de Janeiro, Av. Carlos Chagas Filho 373, Bloco F Sala F1-26, Rio de Janeiro 21941-902, Brazil; ^3^Laboratório de Farmacologia Molecular, Instituto de Ciências Biomédicas, Universidade Federal do Rio de Janeiro, Av. Carlos Chagas Filho 373, Bloco J Sala J1-10, Rio de Janeiro 21941-902, Brazil

## Abstract

Glioblastoma (GBM) is the most aggressive tumor of the central nervous system, and the identification of the mechanisms underlying the biological basis of GBM aggressiveness is essential to develop new therapies. Due to the low prognosis of GBM treatment, different clinical studies are in course to test the use of histone deacetylase inhibitors (iHDACs) in anticancer cocktails. Here, we seek to investigate the impact of HDAC activity on GBM cell behavior and plasticity by live cell imaging. We pharmacologically knock down HDAC activity using two different inhibitors (TSA and SAHA) in two different tumor cell types: a commercial GBM cell line (U87-MG) and primary tumor (GBM011). Upon 72 hours of *in vitro* iHDAC treatment, GBM cells presented a very unusual elongated cell shape due to tunneling tube formation and independent on TGF-*β* signaling epithelial to mesenchymal transition. Live cell imaging revealed that voltage-sensitive Ca^++^ signaling was disrupted upon HDAC activity blockade. This behavior was coupled to vimentin and connexin 43 gene expression downregulation, suggesting that HDAC activity blockade downgrades GBM aggressiveness mostly due to tumor cell competence and plasticity modulation *in vitro*. To test this hypothesis and access whether iHDACs would modulate tumor cell behavior and plasticity to properly respond to environmental cues *in vivo*, we xenografted GBM oncospheres in the chick developing the neural tube. Remarkably, upon 5 days in the developing neural tube, iHDAC-treated GBM cells ectopically expressed HNK-1, a tumor-suppressor marker tightly correlated to increased survivor of patients. These results describe, for the first time in the literature, the relevance of iHDACs for *in vivo* tumor cell morphology and competence to properly respond to environmental cues. Ultimately, our results highlight the relevance of chromatin remodeling for tumor cell plasticity and shed light on clinical perspectives aiming the epigenome as a relevant therapeutic target for GBM therapy.

## 1. Introduction

Glioblastoma (GBM) is the most aggressive tumor of the central nervous system (CNS). This tumor arises from glial cells and is classified as a grade IV glioma, causing focal or scattered anaplasia and presenting accelerated growth with histological diagnosis based on nuclear atypia and mitotic activity [1]. Despite many advances in research into the treatment of this type of cancer in the last decades, the prognosis is of 25 months after the first medical intervention and there has been an improvement in survival of only 2% in 5 years. GBM has been shown to be resistant to radiotherapy and chemotherapy and invariably occurring following surgical resection followed by chemo/radiotherapy [2]. One of the reasons for GBM resistance to therapeutic intervention is the complexity of the tumor itself, which presents regions of pseudopalisade necrosis, hemorrhage, pleomorphic nuclei/cells, and microvascular proliferation. Indeed, following this line of reasoning, growing evidence indicates that rare populations of tumor cells, called tumor stem cells, play a significant role in GBM resistance mainly contributing to the high degree of phenotypic, cellular, genetic, and epigenetic heterogeneity. Cancer stem cells (CSCs) are crucial to boost invasive tumor growth and subsequent relapse [3].

GBM genetics is characterized by several deletions, amplifications, and point mutations that lead to the activation of different signal transduction pathways [4]. More deeply, epigenetic processes add layers of complexity on cancer biology, increasing heterogeneity, and complexity of tumors and, consequently, decreasing efficacy of treatment [5, 6]. Indeed, due to the low prognosis of GBM treatment, different clinical studies are in course to test the use of inhibitors of histone deacetylase (HDAC) activity in anticancer cocktails [7]. HDAC inhibitors (iHDACs) are among the most successful examples of epigenetic therapy for different types of malignancies, including GBM. In fact, preclinical studies have demonstrated the efficacy of different inhibitors of HDAC activity as antitumor agents, especially when associated with other therapies, including chemotherapy and radiation [8, 9]. Numerous studies have shown that there is a wide variety of iHDACs such as valproic acid (VPA), sodium butyrate, vorinostat, tricostatin A (TSA), panobinostat, and entinostat currently used in medical practice [10, 11]. In addition, several iHDACs are Food and Drug Administration (FDA) approved [12], including Vorinostat [13–16] and VPA [17], which are currently being tested in clinical trials on GBM as either monotherapies or combination therapies.

HDAC enzymes catalyze the removal of the acetyl radicals from the lysine residues of the N-terminal tail of the nucleosomal histones [18], resulting in electrostatic changes favoring chromatin compaction [7]. Due to this fact, the HDAC activity is mainly related to transcriptional repression [19]. In humans, 18 proteins with deacetylase domain are coded in the genome and are highly conserved among eukaryotes. Human HDACs are classified into four different classes: class I (HDACs 1, 2, 3, and 8), class IIa (HDACs 4, 5, 7, and 9), class IIb (HDACs 6 and 10), III (SIRT1–7), and IV (HDAC 11), according to structure similarity, enzymatic function, cell location, and expression pattern. Interestingly, HDACs have been inversely correlated to overall survival rates, also presenting a tight correlation with a poor prognosis in GBM patients [20]. In addition, it is very well documented that iHDACs also inhibit DNA damage repair response and influence the response of tumor cells to radiation, inducing cell cycle arrest, senescence, and autophagy [21]. For these reasons, iHDACs have emerged as excellent therapeutic targets for GBM therapy and, regardless all advances described above, the exact mechanisms for iHDACs are still poorly understood.

In this work, we uncovered a new aspect of iHDAC action at the cellular level in real time by time-lapse video microscopy and xenografts in the chick developing neural tube. We found that, after iHDAC treatment, GBM cells presented a very unusual elongated cell shape due to formation of actin- and/or tubulin-rich tunneling tubes in an independent epithelial to mesenchymal transition (EMT) fashion. HDAC activity was also necessary for GBM cell cycle progression, viability, and migration. This iHDAC-induced switch in tumor cell shape due to tunneling tube formation was followed by vimentin and connexin 43 gene expression downregulation, an increase in radio sensitization, and intracellular Ca^2+^ signaling disruption. When GBM oncospheres were placed in the developing neural tube, iHDAC-treated GBM cells expressed HNK-1, a molecular marker tightly correlated to tumor suppression and increased survival of patients. To date, this is the first report on HDAC-dependent HNK-1 expression in GBM cells. Our results describe the relevance of iHDACs for tumor cell morphology and competence highlighting the relevance of chromatin remodeling for tumor cell plasticity. These results shed light on clinical perspectives aiming the epigenome as a relevant therapeutic target for GBM therapy.

## 2. Materials and Methods

### 2.1. Cell Culture

GBM11 cells were isolated from a surgical biopsy of a 57-year-old male diagnosed with glioblastoma. The procedure was performed at the University Hospital of Clementino Fraga Filho (HUCFF) and was approved under the code Conselho Nacional de Saúde (CONEP) No. 2340 as previously described [22]. The GBM cell line U87-MG was obtained from the American tissue culture collection (ATCC) and properly genotyped and certified by the Laboratory of Macromolecular Metabolism Firmino Torres de Castro at UFRJ. GBM cells were cultured in 24-well plates at 2.5 × 10^5^ cells with DMEM (low glucose), supplemented with 10% fetal bovine serum (FBS) and penicillin/streptomycin (PS). Cultures were maintained at 37°C and with 5% CO_2_.

### 2.2. Drug Treatments

At 24 hours after seeding, U87-MG cells were treated with 100 nM TSA (Sigma T1952) or 500 nM SAHA (Sigma SML0061) or SB-431542 (Sigma S4317, diluted in DMSO) or DMSO, as the control group, for 72 h. GBM011 cells were treated with 200 nM TSA or 1 *μ*M SAHA.

### 2.3. 3D Cell Culture

For the generation of oncospheres for morphometric analysis, sterile 96-well round bottom plates were pretreated with 1% agarose and the cells were cultured as described above for 72 or 144 hours. The wells containing oncospheres were photographed every 24 hours under an Olympus CKX41 inverted microscope. For the generation of oncospheres for xenografts, we used 1 × 10^6^ U87-MG cells in suspension in 1 ml of culture medium on nonadherent sterile Petri dishes (60 mm) in the presence of 100 nM TSA or DMSO as control.

### 2.4. Morphometric Analysis of Oncospheres

The analyses were performed using the free AnaSP® software as previously described [23]. The area of each oncosphere was segmented to acquire an image in black and white binary mask format so that the morphological parameters were automatically extracted from each analyzed oncosphere. Three oncospheres of each condition were analyzed in the respective treatment times. The cultures were photographed after 72 hours of treatment to measure the diameter of the oncospheres. ImageJ software (https://imagej.nih.gov/ij/index.html) was used for image processing.

### 2.5. Xenograft in Chicken Embryo

Upon 72 hours, control and iHDAC (100 nM TSA) treated oncospheres were grafted into the neural tube lumen or on the neuroepithelium wall in the prosencephalic region of the developing chicken embryo at different embryonic stages ranging from 7 to 12 somites as previously described by our group [24]. The window opened on the egg was sealed with adhesive tape and incubated at 37°C for 5 days. At the end of this period, the eggs were opened and the embryos were removed and washed in 1x PBS. Embryonic membranes were removed, and the embryos were fixed in Fornoy (10% glacial acetic acid, 60% absolute ethanol, and 30% formol) for at least 24 hours. During the dehydration for histological processing, the embryos were photographed. The *in situ* hybridization and HNK-1 immunostaining were performed as previously described by our group [24].

### 2.6. Transwell Migration Assay

Comparative migration experiments were conducted using a conventional 24-well Transwell system (6.5 mm Transwell® (#3422), Corning, NY, USA) with each well separated by a microporous polycarbonate membrane (10 *µ*m thickness; 8 *µ*m pores) into an upper (“insert”) and a lower chamber (“well”). U87-MG and GBM011 cells were plated as described above. Nutritional deprivation of the cells was induced by removing the medium with FBS and introducing the supplemental medium of 0.1% BSA and drugs. After 48 h under treatment, the cells were trypsinized, counted, and plated at the density of 10^5^ cells/insert with medium supplemented with 0.1% BSA. In the lower bottom of the transwell, 600 *µ*l of medium supplemented with 10% FBS was added. As a negative control was used SFB-free medium in the lower bottom of the well. In this way, cells completed 72 h of the treatment in the transwell. After 72 h, cells were fixed using 4% paraformaldehyde and stained with 1% of crystal violet for 10 minutes. Cells on the top surface of the insert were removed with a cotton swab. To quantify the cells that migrated and adhered to the underside of the membrane, five random fields per condition were photographed under an inverted optical microscope using a 10x magnification. A method of quantitation was performed as described by [25]. Migration was determined by calculating the average pixel/area of the five fields in triplicate.

### 2.7. Video Microscopy and FibrilTool Analysis

U87-MG cells were plated and treated as described above and transferred to a culture chamber under controlled conditions of CO_2_ and temperature (5% and 37°C, respectively). The culture chamber was adapted to a Nikon Eclipse TE300 inverted microscope (Nikon, USA). Upon 72 hours, phase contrast images, from the same field of each experimental condition, were captured every minute by using a Hamamatsu C2400 CCD camera (Hamamatsu, Japan). After assembly of the frames, cell motility was quantified. Cells under the same video microscopy conditions were also cultured in coverslips for F-actin immunostaining, and the images were acquired in a confocal microscope at a 60x magnification and analyzed using the FibrilTool Plugin (ImageJ/National Institutes of Health, USA) [26]. For proliferation analyses, each cell performing mitosis was marked with a black dot using the ImageJ program. Then, the total number of dots for each image in the movie was counted and recorded in a table. Thus, it was possible to determine the number of dividing cells as a function of time, for each experimental situation. For cell motility analyses, dots were also marked to follow the position of a chosen cell over time. The trajectory of each marked cell was then tracked. Knowing the total time of the film (72 h) and the cell displacement in that period, it was possible to determine the cell velocity. At least 12 cells were followed, and a mean velocity value was determined for each experimental condition. The results were plotted using GraphPad Prism 6.0 software.

### 2.8. Conventional PCR

U87-MG and GBM011 cells were harvested, and their mRNAs were extracted with TRIzol (Life Technologies), precipitated in ethanol, and reverse transcribed using random hexamers. Qualitative conventional reverse-transcription polymerase chain reaction (PCR) with GoTaq DNA Polymerase was performed following the manufacturer's instructions (Promega). mRNA levels were standardized by parallel PCR using primers to the housekeeping gene, GAPDH (IDT).

### 2.9. Primers

  GAPDH

F: ACCACAGTCCATGCCATCAC; R: TCCACCACCCTGTTGCTGTA

  HDAC 1:

F: ACCGGGCAACGTTACGAAT; R: CTATCAAAGGACACGCCAAGTG

  HDAC 2:

F: TCATTGGAAAATTGACAGCATAGT; R: CATGGTGATGGTGGTGAAGAAG

  HDAC 3:

F: TTGAGTTCTGCTCGCGTTACA; R: CCCAGTTAATGGCAATATCACAGAT

  HDAC 4:

F: ATTCTGAACCACTGCATTTCCA; R: GGTGGTTATAGGAGGTCGACACT

  HDAC 5:

F: TTGGAGACGTGGAGTACCTTACAG; R: GACTAGGACCACATCAGGTGAGAAC

  HDAC 6:

F: TGGCTATTGCATGTTCAACCA; R: GTCGAAGGTGAACTGTGTTCCT

  HDAC 7:

F: CTGCATTGGAGGAATGAAGCT; R: CTGGCACAGCGGATGTTTG

  HDAC 8:

F: TCCCGAGTATGTCAGTATATATGA; R: GCTTCAATCAAAGAATGCACCAT

  Vimentin:

F: GCACATTCGAGCAAAGACAG; R: GAGGGCTCCTAGCGGTTTAG

  E-cadherin:

F: TGCCCAGAAAATGAAAAAGG; R: GTGTATGTGGCAATGCGTTC;

  Actin smooth muscle:

F: AATGGCTCTGGGCTCTGTAA; R: TGGTGATGATGCCATGTTCT;

  Connexin 43:

F: TGGATTCAGCTTGAGTGCTG; R: TCTTTCCCTTAACCCGATCC;

  P2X7:

F: GCAGCTGCAGTGATGTTTTC; R: CACCTCTGCTATCCCCTTCA;

  CACNA1H T-type calcium:

F: CTGTCACTCATGGGCATCAC; R: ATGAAAAGAAGGCCCAGGTT

### 2.10. Flow Cytometry

U87-MG and GBM011 glioblastoma cells were treated as described above, and after 72 hours in culture, cells were harvested by trypsinization and collected for flow cytometry analysis. For the detection of intracellular antibodies, cells were fixed with 2% paraformaldehyde for 30 min, permeabilized with PBS + 1% BSA + 0.2% saponin for 15 min, stained with primary antibody, anti-Nodal (Santa Cruz Antibodies, polyclonal, sc-28913, 1 : 200) or anti-H4K16ac (Millipore, polyclonal, 07–329; 1 : 200), for 30 min, and diluted in PBS + 0.2% saponin solution. Cells were then washed once with PBS + 0.2% saponin and incubated with secondary antibody Alexa conjugated with fluorochrome 647 (Thermo Fisher Scientific, A-21443, 1 : 1000) or conjugated with fluorochrome 488 (Abcam, ab150077, 1 : 1000) and diluted in PBS + 0.2% saponin solution for 30 min. Cells were then washed twice with PBS + 0.2% saponin, resuspended in PBS, and transferred to FACS reading tubes. All samples were kept in ice and protected from the light. A total of 200.000 cells were acquired on a FACSCanto flow cytometer (BD Biosciences) and analyzed using FACSDiva software (version 8.0.1). Cell death was measured with annexin V-FITC apoptosis detection kit (BD Biosciences) according to the manufacturer. Cell cycle analysis by quantitation of DNA content was performed according to Vindelov's protocol (Vindelov, 1985). Briefly, GBM cells were resuspended in 400 *μ*l propidium iodide solution (PBS, 0.1% Triton X-100, 0.1% RNAse, and 50 *μ*g/ml propidium iodide) and incubated on ice for 15 min. Subsequently, cells were analyzed by flow cytometry, using a FACSCanto (BD Biosciences) operated by FACSDiva software, and at least 20,000 events were collected per sample. Cell doublets were gated out using FSC-A vs FSC-H profiles. All data were analyzed using FACSDiva software (Version 8.0.1).

### 2.11. Ionizing Radiation and Cell Proliferation Assay

U87-MG and GBM011 cells were treated for 72 h as described above and irradiated with 10 Gy in a single dose using a linear 6 MV accelerator with 25 × 25 cm^2^ equivalent field adjusted to a window size and surface distance of 70 cm. After irradiation, MTT assays and flow cytometry were performed at 24 h, 48 h, and 72 h points, and MTT absorbance was read at 570 nm. Cell morphology was evaluated by bright-field and fluorescence microscopy with Falloidin 546 plus DAPI. The images were obtained under a Leica TCS SP5 AOBS confocal microscope.

### 2.12. Fluorimetry Assay

We have used Fluo-4 AM and Fura-2 AM (Molecular Probes F14201) to measure the intracellular increase in Ca^2+^ in U87-MG and GBM011 cells. U87-MG and GBM011 cells were plated as described above in round 15 mm coverslips (Fisher-545-102) coated with poly-L-lysine (Sigma P-2636) and treated with DMSO and iHDACs as described above for 72 hours. The culture medium was replaced by 1 ml of culture medium (DMEM) containing 1 mM probenecid, 0.04% pluoronic, 2 *μ*M Fluo-4 AM (Molecular Probes), or 4 *μ*M Fura 2-AM (to analyze the basal level of intracellular calcium by fluorescence intensity). Cells were incubated at 37°C for 45 minutes, washed with fluorimetry solution (145 mM NaCl, 5 mM KCl, 1.2 mM Na_2_HPO_4_, 4 mM CaCl_2_, 1 mM MgCl_2_, 5 mM HEPES, and 10 mM d-glucose) and transferred to a chamber (P-5 Platform, Warner Instruments, Hamden, CT) with perfusion and a capacity of 200 *μ*l. The fluorescence of 100 cells was continuously monitored for approximately 300 seconds in a fluorescence microscope (Eclipse Ti-U; Nikon). Cells were continuously perfused with fluorimetry solution and stimulated with different solutions (20 mM potassium chloride and 1 mM ATP for Fluo-4 AM). The solutions were added to the cells by using a gravity infusion system, and the variation of intracellular Ca^++^([Ca^2+^]I), was evaluated by the fluorescence emitted at 488 nm using a lambda DG4 illumination system (Sutter Instrument, Novato, CA, on a 40x objective and 510 nm band-pass filter (Semrock, Rochester, NY). The data were acquired using the software MetaFluor (Molecular Devices, Sunnyvale, CA). The Fluo-4 AM data were processed by the software ClampFit (version 10.7.0.3, Molecular Devices). The Fura-2 AM data were processed by the software ImageJ, and the basal levels of intracellular calcium were assessed by fluorescence intensity (Integrated Density).

### 2.13. Statistical Analysis

GraphPad Prism (v6.0, La Jolla, CA) was used for ordinary one-way or two-way ANOVA analysis. If the ANOVA produced a significant result, post hoc pairwise comparisons were tested for significance in which the *P* value was adjusted (*P*_adj_ < 0.05) by Tukey's method for multiple comparisons inside each group and by Sidak's method for multiple comparisons among the individual groups. Results are presented as mean ± SD or mean ± SE, and statistical relevance was defined as *P* < 0.05.

## 3. Results

### 3.1. SAHA Treatment Leads to GBM Cell Cycle Arrest in G0/G1 and Decreased Cell Viability

Since HDACs have been reported to be expressed in GBM cells [28], we first performed a conventional PCR to detect the expression pattern of HDACs in commercial U87-MG and GBM011 primary tumor cells in culture. We found that HDACs 1–7 were constitutively expressed by U87-MG cells and HDACs 1–5 and 7 were constitutively expressed by GBM011 (Supplementary Figure S1(A, A')). We also performed flow cytometry to detect histone hyperacetylation upon iHDAC treatment. Indeed, upon 72 h of iHDAC treatment, there was an increase in the frequency of H4K16ac + U87-MG cells followed by a significant increase in fluorescence intensity per cell (Supplementary Figure S1(B-E)). These first results show that GBM cells express HDACs from classes I, IIa, and IIb, whose activity blockade leads to a hyperacetylated chromatin status.

Next, we monitored the effects of iHDAC treatment along the 72 hours of treatment. Here, we will be comparing the effects of two different iHDCAs, TSA and SAHA. While TSA is one of the most used iHDACs in experimental approaches, SAHA (Varinostat) has already been approved for clinical use [29]. Both U87-MG and GBM011 cells presented a similar behavior during cell cycle progression with respect to TSA treatment. The percentage of cells observed in the G0/G1 phase was similar to that of control groups along the time in TSA-treated cells (Supplementary Figure S2(A-E)). In contrast, HDAC activity was differentially necessary for cell progression when SAHA was added to the medium. While the percentage of U87-MG cells in the G0/G1 phase drastically decreased after 72 h of SAHA treatment, only a discrete decrease was observed in GBM011 cells (Supplementary Figure S2(B-F)). In agreement, cell viability was mostly similar between U87-MG and GBM011 upon TSA treatment (Supplementary Figure S3). Moreover, both U87-MG and GBM011 presented significant decrease in cell viability upon SAHA treatment (Supplementary Figure S3). We conclude that U87-MG and GBM011 present a similar response to TSA during cell cycle progression and cell viability maintenance. In contrast, cell cycle and viability were drastically affected by SAHA treatment in U87-MG while GBM011 was less affected.

Live cell imaging supports a key role for HDAC blockade during tunneling tube formation through F-actin cytoskeleton stabilization in glioblastoma cells.

**Figure 1:**
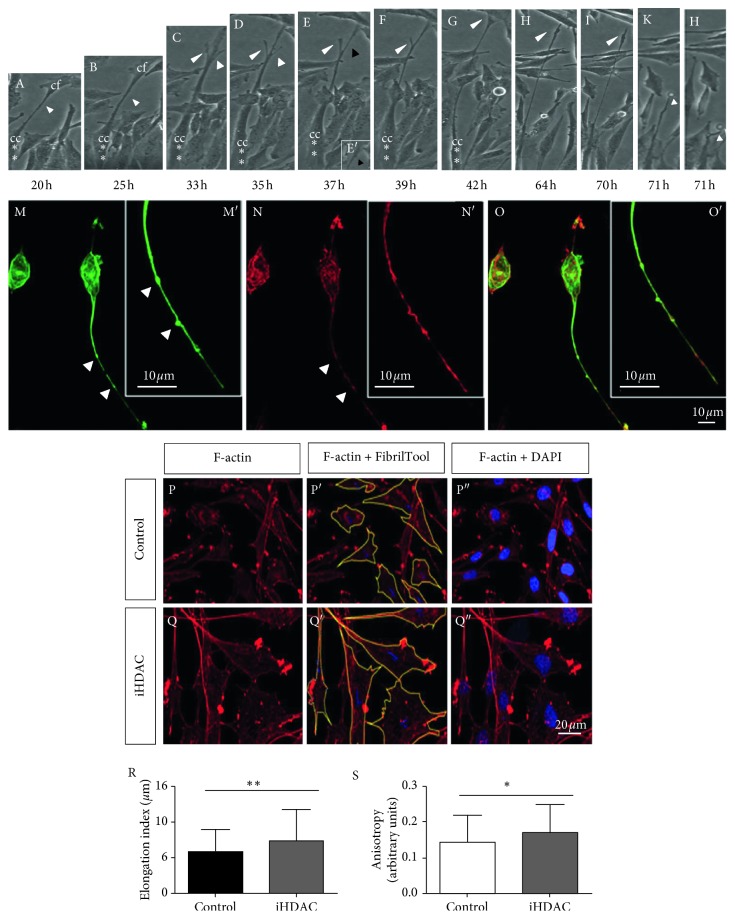
HDAC blockade leads to tunneling tube formation through F-actin cytoskeleton stabilization. (A–L) Time-lapse video microscopy of iHDAC-treated U87-MG cells during 72 h identified the formation of tunneling tubes starting at 20 h after treatment (A, white arrow; red asterisks evidence cell body). Membrane extensions of one cell touch the cell body of neighboring cells, interconnecting tumor cells starting at 35 h after treatment (D). At this time point, transitory membrane protrusions can be noticed (D, white head arrows). As the cell body migrates, it elongates (D, red asterisk) and the transitory membrane protrusion is retracted (E, black head arrow) at the same time that it is possible to note the formation of gondolas along the tunneling tube (E′, black head arrow). The tunneling tube now reaches a neighboring cell (E–G). After losing contact with the neighboring cell, the membrane filament completely retracts (H–L, white head arrow). It was possible to estimate that the membrane filament remained stretched for about 6 hours after loss of contact with the neighboring cell (I–L). iHDAC-treated cells presented type I (actin rich) and type II nanotubes (actin and tubulin rich) which also harbor gondolas (M–O, white head arrows). The elongation index was measured and iHDAC-treated cells (Q, R) were more elongated than DMSO group (P, R). Nanotubes formed in iHDAC-treated cells were more stable and organized than in the control group as found upon F-actin fibrillar structure and anisotropy analysis (S).

**Figure 2:**
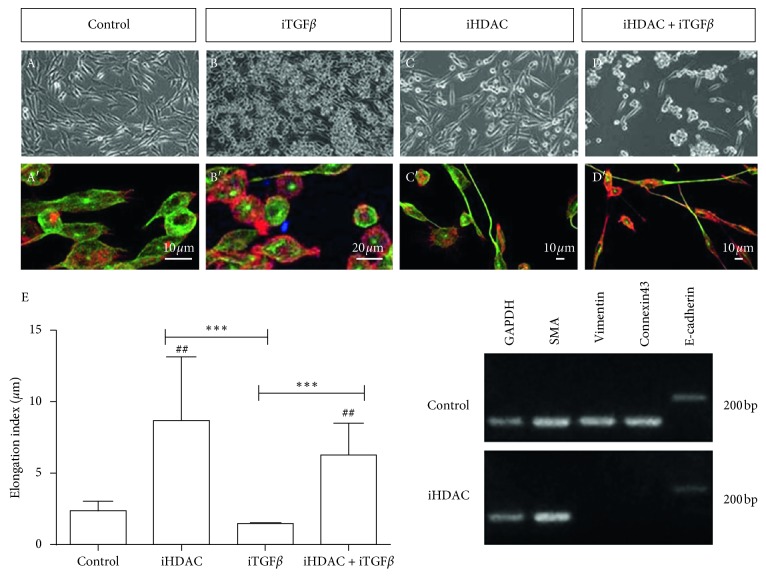
HDAC activity blockade leads to tumor cell shape changes in parallel to EMT and TGF-*β* signaling pathway through tunneling tube formation. U87-MG cells were treated with iTGF-*β* (25 *μ*M SB-431542) in the presence or absence of iHDACs (100 nM TSA). Confocal microscopy characterized type I (C′, actin-rich nanotubes) and type II nanotubes (C′, actin- and tubulin-rich nanotubes) in iHDAC-treated cells. Elongation index showed that iHDAC + iTGF-*β*-treated cells displayed the same elongation index as that of iHDAC-treated cells (E). Qualitative RT-PCR performed with U87-MG cells treated with TSA 100 nM for 72 hours revealed that vimentin and connexin 43 expression was suppressed upon TSA treatment. Asterisks in F indicate comparisons made within the same experimental group at 24 h. *n*=3, different experiments. ^*∗*^ *p* < 0.05; ^*∗∗*^ *p* < 0.01; ^*∗∗∗*^ *p* < 0.001.

However, upon 72 h hours of iHDAC treatment, we noticed an atypical elongated cell shape only in iHDAC-treated GBM cells. These changes in tumor cell shape were evidenced by time-lapse video microscopy and corroborated by morphometric analysis. Using time-lapse video microscopy, we observed in TSA-treated U87-MG cells that the membrane extensions of one cell touch the cell body of neighboring cells, interconnecting tumor cells (Supplementary Video S1, DMSO; Supplementary Video S2, iHDACs; Figure 1(D-G), white head arrow). As the cell body migrates, the established focal contact is stabilized causing a stretching of the cell membrane, resembling an elastic (Figure 1(A, B, D–H, M–O)). It is also possible to notice two active lateral membrane projections from the central (longer) one that are not stabilized and retract (Figure 1(C–E)). After losing contact with the neighboring cell, the membrane filament completely retracts (Figure 1(I–L)). It was possible to estimate that the membrane filament remained stretched for about 6 hours after loss of contact with the neighboring cell (Figure 1(E-H). In fact, morphometric analysis showed that iHDACs evoked a cell shape transition in both U87-MG (Figure 1(P)) and GBM011 tumor cells (Supplementary Figure S4). The elongation index was significantly higher in TSA-treated U87-MG cells than in the control group (Figure 1(R)). In addition, the anisotropy of TSA-treated U87-MG (Figure 1(S)) and GBM cells (Supplementary Figure S4) was higher than in the control group showing that HDAC activity is relevant to maintain cytoskeletal organization. Due to the similarity between the morphology of GBM cells upon iHDAC treatment and the morphological aspects conferred by epithelial to mesenchymal transition (ETM), we decided to test whether iHDAC would be modulating ETM in GBM cells. It has been shown that TGF-*β*1 signaling pathway plays an essential role in the EMT processes, leading to morphological changes closely related to the mesenchymal morphology [30]. In this sense, we decided to test whether the blockade of HDAC activity could regulate EMT by blocking the TGF-*β* signaling pathway with 25 *μ*M SB-431542 (TGF-*β*i) in the presence of iHDAC. U87-MG cells were monitored for cell shape changes upon 72 h of TSA treatment. Interestingly, we noticed that the elongation index of iHDAC + iTGF-*β* treated cells did not present significant difference when compared to iHDACs (Figure 2(A-E)). These results indicate that HDAC activity is not at downstream to TGF-*β* signaling during tumor cell elongation. In addition, TSA treatment led to a suppression in the expression of vimentin, while smooth muscle actin (SMA) and E-cadherin were still constitutively expressed by U87-MG cells even upon TSA treatment (Figure 2(F)). In addition, TSA treatment led to suppression of connexin 43 expression, suggesting that cell coupling by gap junctions was also disrupted by HDAC activity blockade. Thus, this data set excludes the hypothesis that EMT is being modulated by the blockade of HDAC activity and strongly suggests that the observed morphological differences between the groups are due to the structuring of tunneling tubes. Taking into account the features described above, we characterized type I and type II nanotubes in TSA-treated U87-MG cells. Type II nanotubes started growing as cells moved apart; they were enriched in actin and tubulin and harbored dilatations of the tube forming a gondola (Figure 1(M-O)). In fact, as observed by live cell imaging, nanotubes formed in iHDAC-treated GBM cells were more stable and organized than in the control group as found upon F-actin fibrillar structure and anisotropy analysis.

**Figure 3:**
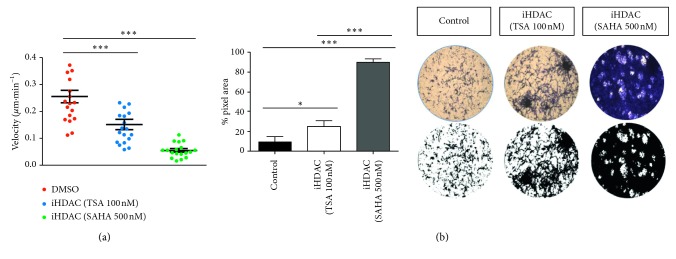
HDAC activity is required for the GBM migration velocity. U87-MG cells were treated for 72 hours with TSA or SAHA and were monitored by time-lapse video microscopy or submitted to the transwell assay. Treatment with TSA led to a significant decrease in the rate of cell migration that was corroborated by the transwell assay. It is possible to observe a larger number of cells retained in the transwell matrix in the TSA group than in the control group. The same effect was observed when the cells were treated with SAHA, but this effect was enhanced when compared to TSA. *n*=3. ^*∗∗∗*^ *p* < 0.001; ^*∗∗∗∗*^ *p* < 0.0001.

Next, we examined the effects of iHDAC treatment on GBM cell motility by time-lapse video microscopy and transwell migration assay. Upon 72 h of iHDAC treatment, U87-MG GBM cells presented a decrease in the velocity of migration that was corroborated by transwell assay. We detected a higher number of tumor cells retained in the transwell matrix after 72 hours when compared to the control group (Figure 3). This behavior was enhanced in the SAHA-treated group when compared to either the TSA or control group (Figure 3). In contrast, GBM011 cells did not present significative differences in cell velocity and migration to the control group when treated with TSA or SAHA (Supplementary Figure S5).

Thus, we conclude that HDAC activity blockade led to tumor cell shape changes through tunneling tube formation. We also conclude that tumor malignancy was downgraded because migration velocity was decreased, what may impact on tumor dissemination and infiltration.

### 3.2. HDAC Activity Is Necessary for GBM Radioresistance

**Figure 4:**
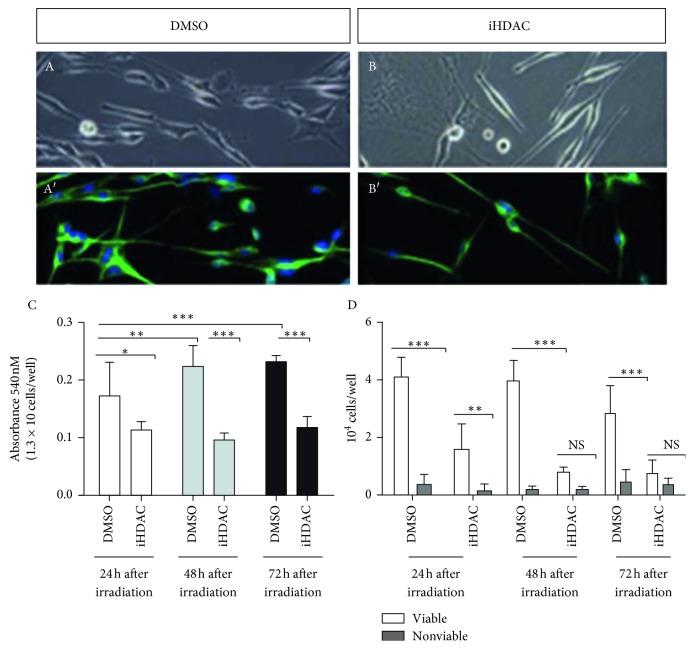
iHDAC treatment radiosensitizes U87-MG cells that, in turn, increase mitochondrial activity. U87-MG cells were irradiated (A, B), and MTT assay showed a significant increase in absorbance in the DMSO group at 24 h and 48 h after ionizing irradiation (C) and did not show significant differences, at the same time points, in iHDAC-treated cells (C). Cell counting using trypan blue at 24 h, 48 h, and 72 h time points after irradiation showed a significant decrease in cellular viability between the DMSO and iHDAC groups (D). *n*=9; ^*∗*^ *p* < 0.05; ^*∗∗*^ *p* < 0.01; ^*∗∗∗*^ *p* < 0.001.

According to the literature, GBM has been shown to be resistant to radiotherapy and invariably reoccurring after surgical resection. Previous work from the literature has also clearly demonstrated the relevance of iHDACs to increase GBM radiosensitization [11]. Thus, we compared the response of GBM cells to radiotherapy upon iHDAC treatment. U87-MG treated with TSA for 72 h showed a significant difference in the number of viable cells between control and TSA-treated cells (Figure 4(D, E)). In fact, we observed that the TSA-treated group showed no significant increase in absorbance at 24 h and 48 h after irradiation, indicating that the treatment radiosensitized tumor cells (Figure 4(D, E)). In contrast to U87-MG cells, GBM011 cells still constitutively expressed SMA, vimentin, connexin 43, and E-cadherin when treated with TSA or SAHA (Supplementary Figure S6(A)). In fact, GBM011 cells treated with TSA did not present consistent differences in cell viability when compared to the control group 72 hours after irradiation (Supplementary Figure S6(C, E, G)). SAHA-treated GBM011, however, presented significative decrease in cell viability 72 h after irradiation (Supplementary Figure S6). We propose that HDAC-mediated radiosensitization of GBM cells is due to vimentin and connexin 43 expression downregulation.

### 3.3. Live Imaging of HDAC Blockade Uncovers Voltage-Sensitive Ca^++^ Signaling Disruption in GBM Cells

**Figure 5:**
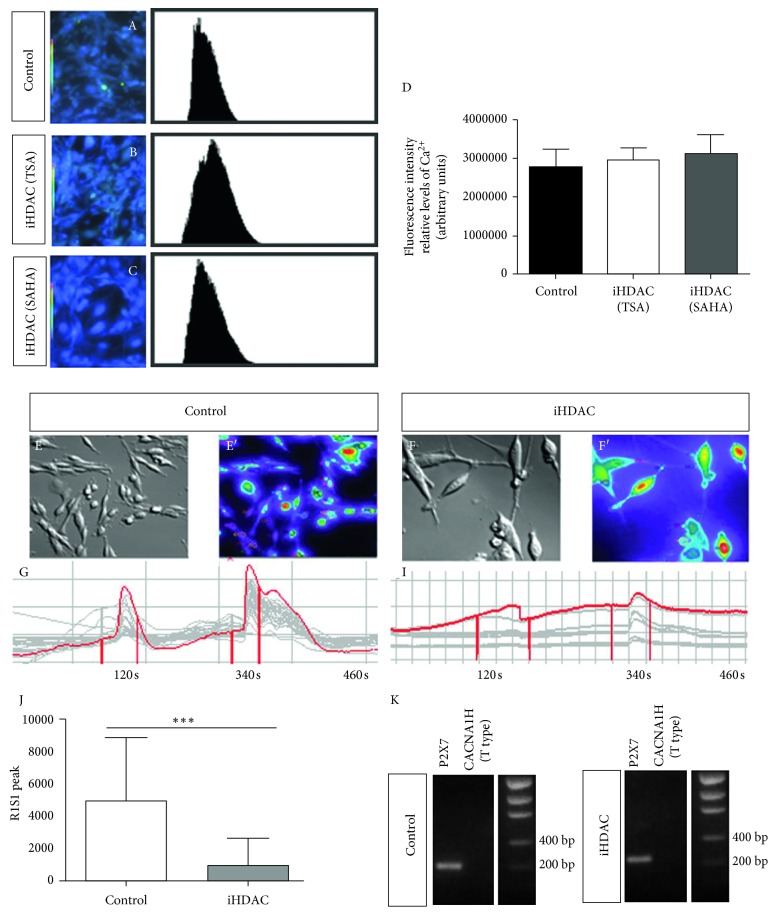
iHDAC treatment disrupts voltage-dependent Ca^++^ channels signaling by live cell imaging. iHDAC-treated U87-MG cells (100 nM TSA for 72 hours) were incubated with Fura 2-AM and analyzed by confocal microscopy. Significative differences in the basal levels of intracellular Ca^2+^ were not detected among the experimental groups (A–D). The fluorescence of 100 cells was continuously monitored for approximately 10 minutes in a fluorescence microscope (E-F). DMSO (E–G) or iHDAC (F–I) treated cells were continuously perfused with fluorimetry solution and stimulated with 20 mM KCl and 50 mM ATP. The variation of intracellular Ca^++^ ([Ca^2+^]I) was evaluated by the fluorescence emitted at 488 nm. The responsiveness to KCL and ATP was quantified, and U87-MG cells treated with iHDACs were less responsive to KCl and ATP than DMSO-treated cells (J). Qualitative RT-PCR revealed constitutive expression of P2X7 in both control and iHDAC-treated groups. On the contrary, the alpha subunit 1H of the CACNA T-type calcium channel is not expressed by U87-MG cells. ^*∗∗∗*^ *p* < 0.001.

To test the functional status of GBM cells under iHDAC treatment, we investigated the efficiency of tumor cells in transducing Ca^2+^ signal through the cell membrane. Several studies have shown that intracellular calcium ([Ca^2+^]I) concentration can significantly contribute to cell biochemical mitotic signaling, migration, apoptosis, cell cycle control, and cell volume regulation, all of them critical for cell survival and proliferation [31]. The main types of Ca^2+^ channels described in the literature and expressed by gliomas are voltage-dependent channels (Ca_v_1 = Type L; Ca_v_2 = Type P/Q, R, N; Ca_v_3 = Type T) and purinergic receptors [32, 33]. Several studies suggest that the P2X7 receptor, the purinergic ionotropic receptor (P2X7R), and Type T low voltage play an important role in GBM behavior including glioma progression [34, 35]. Thus, we sought to better understand the biological significance of iHDACs in the context of Ca^2+^ signaling transduction. After 72 hours, the basal levels of intracellular Ca^2+^ were assessed by confocal microscopy in U87-MG and GBM011 cells under TSA and SAHA treatment. While significative differences in basal levels of intracellular Ca^2+^ were not detected in U87-MG cells (Figure 5(A)), GBM011 treated with SAHA presented a significative decrease (Supplementary Figure S7). Our results showed that control cells were responsive to both stimuli applied, KCl (20 mM and 50 mM), a depolarizing agent that acts on low-voltage channels, and ATP, an agonist that binds to that receptor and leads to cell depolarization. Quantitative analyses of [Ca^2+^]I showed that the control group presented a higher response to the stimuli than U87-MG cells treated with TSA (Figure 5(E-J)). In addition, control and TSA-treated U87-MG cells constitutively express P2X7 but do not express the alpha subunit 1H of the T-type calcium channel (Figure 5(K)). We conclude that iHDAC treatment disrupts intracellular Ca^2+^ upon stimulus even in the presence of similar basal levels of intracellular Ca^2+^ when compared to the control group.

### 3.4. HDAC Activity Blockade Downgrades GBM Malignance and Makes Tumor Cells Competent to Properly Respond to Environmental Cues In Vivo

**Figure 6:**
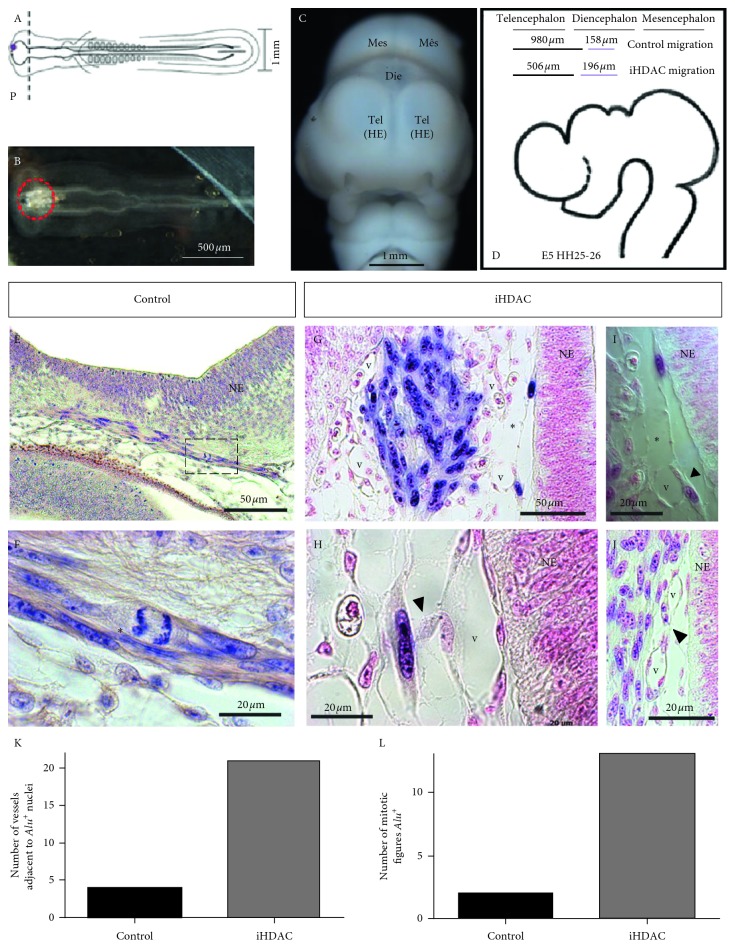
HDAC activity blockade in synergy with the developing embryo leads to tunneling tube formation in U87-MG cells. U87-MG oncospheres were generated *in vitro,* exposed to iHDACs (100 nM TSA) for 72 hours prior xenogaft and placed on the neuroepithelium wall (A) or inside the developing neural tube at the prosencephalic level (B). At E5 chick developmental stage (C), embryos were fixed and *in situ* hybridization was performed using *Alu* probe. *In situ* hybridization revealed that both control and iHDAC-treated U87-MG cells were integrated into the embryonic mesenchyme. iHDAC-treated cells spread for longer distances than control cells (D). The embryonic environment efficiently promoted cell proliferation in both control and iHDAC-treated cells (E and F, red asterisks), bypassing iHDAC inhibition of cell proliferation observed *in vitro* as more mitotic figures *Alu* ^*+*^ were found in the iHDAC group (K). Tunneling tube formation was observed *in vivo* only in iHDAC-treated cells (G and I, asterisks). Tunneling tubes connected neighboring tumor cells (G and I, asterisks) as well as tumor cells and embryonic vessels (H, arrow head). More embryonic vessels were found close to *Alu* ^*+*^ nuclei in iHDAC-treated cells than in control cells (J, L). NE = neuroepithelium; V = embryonic vessel.

Taken together, our results show that iHDAC treatment leads to tunneling tube formation and tumor malignance downgrade *in vitro.* Our interpretation relies on the fact that iHDAC-treated cells decrease the velocity of migration and downregulate vimentin and connexin 43 expression, and to test whether TSA-treated U87-MG cells in fact harbor the competence to properly respond to environmental cues and downregulate their malignant behavior, we used GBM oncospheres xenografted in the developing neural tube of the chick embryo. Oncospheres containing 1.5 × 10^3^ cells were generated for the study of the morphometric properties after iHDAC treatment. Such oncospheres did not exceed 150 *μ*m in diameter at the end of 4 days in culture (Supplementary Figure S8(A–C)), and iHDAC-treated oncospheres presented decreased diameter and volume when compared to control oncospheres (Supplementary Figure S8(D)). When cell viability was assessed by MTT assay, we did not notice significant differences between control and TSA-treated oncospheres upon 72 hours (Supplementary Figure S8(F)). However, we were able to observe a significant decrease in cell viability at 96, 120, and 144 hours of prolonged treatment (Supplementary Figure S8(E)). This characterization is very relevant as upon xenograft, oncospheres will not be in contact with TSA anymore. For this reason, we monitored the morphometric properties upon TSA removal up to 72 hours after TSA had been removed. Despite the differences observed in the diameter and volume between control and TSA-treated oncospheres along the procedure (Supplementary Figure S8(G, H)), we did not notice significative changes in the morphometric parameters of iHDAC-treated oncospheres (Supplementary Figure S8(I, J)). So, we conclude that TSA treatment has a long-term effect even after inhibitor withdrawal. Then, we performed the xenograft of the control and TSA-treated oncospheres in the anterior prosencephalic region on the right wall of the neural tube shortly after its closure and the onset of neurogenesis (Figure 6(A)). Oncospheres were also placed in the neural tube lumen (Figure 6(B)). After 5 days of development (HH25; Figure 6(C)), the embryos were processed for histological analysis and paraffin sections were developed for *in situ* hybridization using the *Alu* probe for identification of the human cells and immune staining for HNK-1. First, in both groups, we observed that tumor cells successfully integrated into the embryonic mesenchyme and migrated over long distances (Figure 6(D)). By examining serial paraffin sections, we observed that TSA-treated cells migrated longer distances than control cells (Figure 6(D)). In fact, TSA-treated cells were found over a larger area than control cells since we found *Alu* ^*+*^ nuclei at 196 *μ*m beyond the xenograft point. In the control group, we were able to find *Alu* ^*+*^ nuclei at 168 *μ*m beyond the xenograft point (Figure 6(D)). This result bypasses the behavior observed *in vitro* since we found that iHDAC-treated cells presented a decrease in cell velocity when compared to the control group in the time-lapse video microscopy and transwell assay. The quantification of mitotic figures (Figure 6(E, F)) indicates that TSA-treated cells proliferated more than the cells in the control group (Figure 6(L)). We also observed that the association with embryonic vessels was greater in the TSA group than in the control group (Figure 6(K)), suggesting that human tumor cells maybe using the embryonic vessels as migratory routes to disperse in the mesenchyme. Interestingly, we also observed the formation of membrane protrusions similar to the ones observed *in vitro* only in the TSA group. These membrane protrusions were observed in the tumor cell-cell contact in migratory routes on the neuroepithelium (Figure 6(G, I)) and also in the contact between tumor cells and embryonic vessels (Figure 6(H, J)). We conclude that TSA-treated cells mostly recapitulated the behavior observed *in vitro* with respect to tunneling tube formation. However, the effects of TSA treatment on the migratory and proliferative behavior of tumor cells were reversed by the embryonic microenvironment.

**Figure 7:**
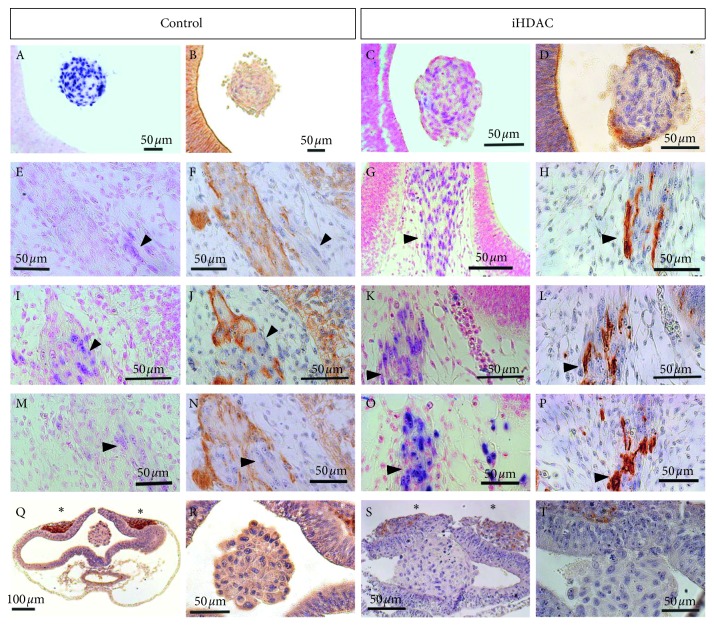
HDAC activity blockade downgrades GBM aggressiveness in synergy with the developing microenvironment. U87-MG oncopheres were cultured in the presence or absence of iHDACs (100 nM TSA) for 72 hours. After the xenograft, the embryos were allowed to develop until stage E5. *In situ* hybridization and immunostaining for HNK-1 revealed that when the oncospheres remained placed in the lumen of the developing neural tube, the outermost layer of cells expressed HNK-1 only in the iHDAC group: control oncospheres: (A) *Alu in situ* hybridization; (B) immunostaining for HNK-1 and iHDAC oncospheres: (C) *Alu in situ* hybridization; (D) immunostaining for HNK-1. The cells that were integrated into the embryonic mesenchyme were able to migrate and to associate with developing embryonic structures. In the control group, only embryonic cells expressed HNK-1 since *Alu* ^*+*^ nuclei did not colocalize with the HNK-1 (E–N, arrow heads). In iHDAC oncospheres, however, it was possible to observe the colocalization of *Alu* ^*+*^ nuclei and HNK-1 staining (G–P, arrow heads). In fact, control (Q, R) and iHDAC (S, T) treated oncospheres did not constitutively express HNK-1 since their placement in the developing neural tube lumen, followed immediately by fixation did not reveal HNK-1 staining in the oncospheres. Positive control for HNK-1 staining was done with migrating neural crest cells (Q–T, asterisks).

We then hypothesized that TSA-treated cells harbor the competence to properly respond to environmental cues and downregulate their malignant behavior as we have mostly observed *in vitro.* To properly approach this question, we performed *in situ* hybridization followed by immune staining in serial sections for the HNK-1 marker, which is widely expressed by neuroepithelial cells and migratory neural crest cells during embryonic neurogenesis. In fact, we were able to detect HNK-1^+^ cells only in the outermost layer of TSA-treated oncospheres placed in the neural tube lumen 5 days after the xenograft (Figure 7(A and B) (control); 7(C and D), TSA). In addition, we found HNK-1^+^ cells dispersed into the embryonic mesenchyme but which were *Alu* ^*−*^, of embryonic origin therefore (Figure 7(E, F, I, J, M, N)). Surprisingly, only in the TSA group, we detected a colocalization of *Alu* ^*+*^ and HNK-1^+^ cells (Figure 7(G, H, K, L, O, P)). Since we observed that oncospheres placed in the lumen of the neural tube and fixed immediately after the xenograft did not express HNK-1 (Figure 7(G, H, K, L, O, P) (control);7(S and T) (TSA)), we concluded that the blockade of HDAC activity confers the competence to the tumor cell to properly respond to environmental cues and downregulate its malignant behavior *in vivo*.

## 4. Discussion

Several studies have reported the pattern of HDAC expression in both health and pathological brain tissue, showing that aberrant expression of HDACs correlates with a poor prognosis in different types of cancer [20]. It has been characterized that there is an increase in the mRNA expression levels of HDAC1, 3, and 6 in both GBM cells and primary GBM tissues [36]. Particularly, several HDAC classes have been reported to be correlated with GBM malignancy and radioresistance [28, 37, 38]. In this scenario, we have identified the HDACs from classes I, IIa, and IIb are expressed by U87-MG cells and primary GBM011 cells whose activity, when inhibited by TSA or SAHA, led to tumor cell shape changes associated with tunneling tube formation which correlated to disruption in the cell cycle and viability, Ca^2+^ responsiveness, and radiosensitization. We also successfully linked this behavior modulated by HDAC activity blockade to the downregulation of vimentin and connexin 43. Interestingly, it was recently reported the correlation among HDACs 4 and 6 and the DNA double-strand break repair machinery towards the maintenance of stemlike phenotype coupled to radioresistance in GBM cells [37]. In this paper, the authors studied samples from 31 GBM patients who underwent temozolomide and radiotherapy, which leads to an enrichment of more undifferentiated, stemlike GBM cells and, therefore, to a poor prognosis. Interestingly, the expression levels of HDAC 4 and HDAC 6 were upregulated in most of the cases and were directly related to a poor clinical prognosis. In addition, molecular ablation of HDACs 4 and 6 radiosensitized U87-MG cells predisposing GBM cells to radiation therapy-induced apoptosis [37]. Following this reasoning, the quantification of HDACs mRNA levels in the normal brain, astrocytoma grades I, II, and III, and GBM did not show significant differences in expression levels of HDACs 6 and 7 [39]. When the comparison was made among low- and high-grade gliomas and normal brain, only HDAC 4 mRNA was upregulated in high-grade gliomas. Comparisons made between GBM and grade III astrocytoma showed that HDACs 4, 6, and 7 were expressed at lower levels in GBM samples. These results suggest that HDACs 4, 6, and 7 expression patterns are restricted to a rare population of GBM cells and may be associated with a more-resistant GBM cell population which may be, at least in part, positively selected by usual radiotherapy and chemotherapy. This interpretation is corroborated by the fact that patients that underwent radiotherapy and chemotherapy presented an enrichment in mRNA levels of HDACs 4 and 6 associated with a stemlike phenotype [37]. These results from the literature corroborate our findings since we have shown that both U87-MG and GBM011 cells express HDACs 4 and 7 (Supplementary Figure S1) whose activity blockade leads to G2/M cell cycle arrest [40] and radiosensitization [37] as we have reported here.

However, our major finding is the report of HDAC-dependent tumor cell shape changes and tunneling tube formation never deeply explored in the literature before by live cell imaging. Plasma membrane protrusions can carry organelles and functionally couple tumor cells establishing a resistant population to radiation-induced cell death also presenting an invasive phenotype [41]. In this paper, the authors showed that tunneling tube formation was connexin 43-dependent and drove cell invasion, proliferation, and radioresistance. However, in contrast, our findings did not corroborate a role for connexin 43 during iHDAC-dependent tunneling tube formation. In fact, connexin 43 was downregulated after TSA treatment and was correlated to radiosensitization of U87-MG cells as previously reported [42]. In contrast, GBM011 cells still express connexin 43 what may contribute to radioresistance observed in TSA-treated GBM011 cells. In addition, we reported a GBM cell shape transition to mesenchymal morphology independent of TGF-*β* signaling and not related to EMT (Figure 2). Thus, once the regulation of EMT by blocking HDAC activity was excluded as a mechanism to promote the elongated morphology, we propose to morphologically characterize the cellular extensions observed in iHDAC-treated cells. To approach this issue, we started analyzing the F-actin fibrillar structure of control and iHDAC-treated cells by live imaging and anisotropy, an index that evaluates the cellular fibrillar organization. In fact, iHDAC-treated U87-MG and GBM011 cells presented a significant higher anisotropy index, indicating a more-organized fibrillar actin structure when compared to the control group (Figure 1). Such organization of the cytoskeleton in iHDAC-treated cells may respond, at least in part, by the elongated and stable morphology observed in the absence of HDAC activity (Figure 1). Dynamic protrusions of cell surfaces are well documented among different cell types and small membrane projections, such as filopodia and microvilli, as well as larger structures, such as lamellipodia, are largely documented in the literature. More recently, thin surface membrane projections have been described as key long-range mediators and named “membrane nanotubes or tunneling tubes” [43]. One interesting function described for nanotubes is focused on their ability to deliver proteins and organelles from one cell to a neighboring cell. Rustom [44] described, for the first time, the transfer of vesicles through nanotubes using neuronal and renal cells. In the context of tumor cell biology, it has been demonstrated that membrane nanotubes form a functional and resistant network to avoid damage caused by radiotherapy [41]. Our results showed that control cells presented an elongated morphology upon radiotherapy, such as those observed in iHDAC-treated cells, suggesting that ionizing radiation modifies the morphology of GBM cells, which are known radioresistant. We also observed that after irradiation, there was an increase in mitochondrial activity, as demonstrated by the MTT, associated with decreased cell viability and total cell number (Figure 4). In fact, although there was a significant difference in the total number of viable and nonviable cells, 24 h after irradiation in the iHDAC group, at 48 h and 72 h after irradiation, it was not possible to observe significant differences.

Another parameter investigated by Osswald et al. [41], also evoked as a resistance mechanism in astrocytomas, was the maintenance of calcium homeostasis through the network communication formed by nanotubes. The authors then suggest that nanotubes are involved in Ca^2+^ signaling spreading from a single cell to neighboring cells. In our study, we aimed to characterize the relevance of Ca^2+^ channels in iHDAC-treated cells. We found that iHDAC cells presented a lower responsiveness to Ca^2+^ stimuli than control cells. Despite the similarities in the basal levels of intracellular Ca^2+^ between control and iHDAC-treated cells, control cells, however, responded to both T-type calcium channels and purinergic receptors (Figure 4). In fact, it has been shown that purinergic receptors (P2X7R) silencing reduces radiotherapy-induced GBM cell cytotoxicity in addition to a less-efficient radiotherapy-induced cell death, demonstrating that high P2X7 expression levels and function are a good prognostic factor for GBM radiosensitivity [34, 35]. In addition, Ca_v_3 (Type T low) voltage-sensitive calcium-channel overexpression was already detected in GBM and pharmacological inhibition resulted in a decrease in cell viability, clonogenic potential, and induction of apoptosis [33].

Thus, our results argue in favor for HDAC blockade as an important hallmark for tumor malignancy downgrade. In fact, when placed in the developing neural tube U87-MG cells successfully integrated into the embryonic mesenchyme, proliferated, and migrated, establishing a close relationship with the embryonic vasculature (Figure 6). The identification of U87-MG cells in nonsequential caudal sections in the iHDAC group suggests the adoption of a possible migratory route. Detachment and the transition to the mesenchymal phenotype contribute to the migratory mechanism [45] and, in this sense, the mesenchymal morphology conferred by HDAC activity blockade may contribute to this kind of behavior. In fact, a recent work using the xenograft model showed that the embryonic microenvironment can orchestrate the cohesion and the detachment of neuroblastoma tumor cells from primary tumors formed in the adrenal medulla after the xenograft was performed in the region corresponding to adrenal sympathetic neural crest. It was possible to infer that there was a recapitulation of the metastatic behavior of these tumor cells both along peripheral nerves and also through the aorta artery during development [46]. In our study, the blockade of HDAC activity led to the formation of a primary aggregate between the telencephalic vesicles, where some cells were also observed, and the migratory process was initiated (Figure 6). In contrast, control U87-MG cells remained adjacent to the neuroepithelium in association with host cells (Figure 6). Finally, the immunostaining for HNK-1 showed that TSA-treated U87-MG cells were positives for this molecular marker. Interestingly, the levels of HNK-1 were found to be inversely proportional to patient survival. In addition, this same work also showed that HNK-1 is an important tumor suppressor for astrocytic tumors [47].

## 5. Conclusions

Thus, our results describe, for the first time in the literature, the relevance of iHDACs for tumor cell morphology, competence, and plasticity through an original approach using live cell imaging coupled to xenografts in the developing chick embryo. Indeed, regardless of our data corroborating with previous work from the literature showing the effects of iHDACs on proliferation, migration, and cell viability, we first connect the biological effects of iHDACs at the cellular level correlating morphological cell changes with functional outcomes, leading GBM cells to decrease the responsiveness to voltage-sensitive Ca^2+^ channels. In addition, we provided evidences that HDAC activity blockade made GBM cells competent to properly interact with the developing microenvironment and downgrade tumor malignancy. Taking into account that GBM stem cells still represent a therapeutic challenge due to the fact that they are resistant to radio and quimiotherapy, the use of iHDACs may represent an interesting strategy to promote GBM stem cell differentiation and consequently sensitization to current strategies used in the clinic. As a consequence, epigenetic mechanism HDAC-mediated may emerge as a tractable approach in GBM therapy and shed light on new clinical strategies for this malignancy.

## Data Availability

All data generated or analyzed during this study are included in this published article or in the supplementary information files.

## Supplementary Materials

Supplementary Materials Supplementary material contains experimental evaluation of HDACs mRNA expression pattern, flow cytometry, immunocytochemistry, conventional PCR, and morphometric analysis of iHDAC-treated oncospheres as follows: Supplementary Figure S1: U87-MG cells and GBM011 primary cells tumor constitutively express HDACs. Supplementary Figure S2: HDAC activity is necessary for cell cycle progression in GBM cells. Supplementary Figure S3: HDAC activity is necessary for GBM cell viability Supplementary Figure S4: HDAC blockade leads to F-actin cytoskeleton stabilization and tunneling tube formation in GBM011 cells. Supplementary Figure S5: HDAC blockade does not disrupt GBM011 migration pattern. Supplementary Figure S6: qualitative RT-PCR showed that GBM011 cells expressed smooth muscle actin (SMA), vimentin, connexin 43, and E-cadherin upon 72 hours of either TSA and SAHA treatment (A) Supplementary Figure S7: HDAC activity blockade disrupts basal levels of intracellular Ca^2+^ in GBM011 cells. Supplementary Figure S8: HDAC activity is necessary for U87-MG morphometric properties.
